# 

**Published:** 1852-08

**Authors:** 


Philadelphia.

### CONTENTS

OF THE

### MEDICAL EXAMINER.

NEW SERIES—NO. XCII.—AUGUST, 1852.

### ORIGINAL COMMUNICATIONS.

Experimental Researches applied to Physiology and Pathology. By E. Brown-Séquard, M. D., of Paris.
On the Source of the Vital Properties, - - - 481
Researches on the Reflex Faculty, - - - - 485
Researches on the Influence of the Nervous System upon the Functions of Organic Life, - - - 486
On the Reparative Power of the Nervous System, - 497
On Turning and Rolling as Phenomena produced by Injuries of the Nervous System, - - - - - 498
On a means of Measuring degrees of Anæsthesia and Hyperæsthesia, - - - - - - 503
Observations on Cholera in Military Practice. By W. H. Tingley, M. D., late an officer of the Medical Staff, U. S. Army, Member of the Academy of Natural Sciences, etc., - - 504
Mortality of Philadelphia, for April, May and June, 1852, arranged from the Record kept at the Health Office. With remarks on the new "Registration Law." By Wilson Jewell, M. D., - 515

Answer to a Friend on Organising the Medical Association. By Samuel Jackson, M. D., formerly of Northumberland, - 527
Case of Acute Metritis treated successfully with Urate of Ammonia. By Dr. C. F. Percival, - - - - - - 531

### BIBLIOGRAPHICAL NOTICES.

Illustrated Manual of Operative Surgery and Surgical Anatomy. By MM. Cl. Bernard and Ch. Huette. Edited with notes and additions, and adapted to the use of the American Medical Student, by W. H. Van Buren, M. D., Surgeon to Bellevue Hospital, &c., and C. E. Isaacs, M. D., Demonstrator of Anatomy in the College of Physicians and Surgeons, New York. Illustrated with steel engravings from drawings after nature, by M. J. Léveillé. Designed to serve as a companion to the ordinary text-books of Surgery, - - - - - 533
The Principles of Surgery. By James Miller, F. R. S. E., F. R. C. S. E., &c. &c. Third American, from the Second and enlarged Edinburgh Edition. Illustrated by two hundred and forty engravings on wood. Revised with additions by F. W. Sargent, M. D., Member of the College of Physicians, Author of Minor Surgery, &c., - - - - - - - 535
An Analysis of Physiology; being a condensed view of the most important facts and doctrines. Designed especially for the use of Students. By John J. Reese, M. D., Lecturer on Materia Medica and Therapeutics in the Medical Institute of Philadelphia, &c. &c., - - - - - - - 535

### EDITORIAL.

Extract from a Review of Dr. Gross's work on the Urinary Organs, - 536
Pirrie's Surgery, - - - - - - - - 536
Death of Dr. James B. Rogers, - - - - - - 537

### MEDICAL NEWS.

Election of Dr. Edward Hartshorne as Surgeon to Wills' Hospital, - 537
Hospital of the Protestant Episcopal Church in Philadelphia, - 537
Appointment of Dr. Joseph Leidy as Pathologist to St. Joseph's Hospital, - - - - - - - - - 537

### RECORD OF MEDICAL SCIENCE.

### PATHOLOGY AND PRACTICE OF MEDICINE.

Pulmonary Emphysema, - - - - - - - 538
Case of Pseudo-Membranous Laryngo-Bronchitis, or Croup, in an Adult. By M. Bouillaud. Clinical Remarks on Laryngo-Bronchial Diphtheritis, or Croup, in Adults. By Dr. Bonnet, 544
Case of Spasmodic Constriction of the Œsophagus. By Dr. B. Palais, 544
On Diphtheritis of the glans in some cases of Paralysis. By Dr. Hérard, - - - - - - - - - 545
Some facts in Pathological Anatomy regarding the state of the spleen in the intermittent fever of Madagascar. By Dr. Rochard, - 545
Obstinate intermittent coryza—instantaneous cure. By D. A. Menudier, - - - - - - - - - 546

### OBSTETRICS.

On Abdominal Tumors during Pregnancy. By Dr. Morisseau, - 546

### SURGERY.

New Mode of Reducing Strangulated Hernia, - - - 547

### MATERIA MEDICA AND THERAPEUTICS.

On the Action of Chloroform. By M. Hervez de Chégoin, - - 548

### CHEMISTRY.

On the Use of Barium, Cadmium and Nickel in Pharmacy, - - 548

